# DeepT3_4: A Hybrid Deep Neural Network Model for the Distinction Between Bacterial Type III and IV Secreted Effectors

**DOI:** 10.3389/fmicb.2021.605782

**Published:** 2021-01-21

**Authors:** Lezheng Yu, Fengjuan Liu, Yizhou Li, Jiesi Luo, Runyu Jing

**Affiliations:** ^1^School of Chemistry and Materials Science, Guizhou Education University, Guiyang, China; ^2^School of Geography and Resources, Guizhou Education University, Guiyang, China; ^3^College of Cybersecurity, Sichuan University, Chengdu, China; ^4^Department of Pharmacology, School of Pharmacy, Southwest Medical University, Luzhou, China

**Keywords:** Gram-negative bacteria, secreted effector, deep learning-artificial neural network, recurrent neural networks, deep neural networks

## Abstract

Gram-negative bacteria can deliver secreted proteins (also known as secreted effectors) directly into host cells through type III secretion system (T3SS), type IV secretion system (T4SS), and type VI secretion system (T6SS) and cause various diseases. These secreted effectors are heavily involved in the interactions between bacteria and host cells, so their identification is crucial for the discovery and development of novel anti-bacterial drugs. It is currently challenging to accurately distinguish type III secreted effectors (T3SEs) and type IV secreted effectors (T4SEs) because neither T3SEs nor T4SEs contain N-terminal signal peptides, and some of these effectors have similar evolutionary conserved profiles and sequence motifs. To address this challenge, we develop a deep learning (DL) approach called DeepT3_4 to correctly classify T3SEs and T4SEs. We generate amino-acid character dictionary and sequence-based features extracted from effector proteins and subsequently implement these features into a hybrid model that integrates recurrent neural networks (RNNs) and deep neural networks (DNNs). After training the model, the hybrid neural network classifies secreted effectors into two different classes with an *accuracy*, *F*-value, and *recall* of over 80.0%. Our approach stands for the first DL approach for the classification of T3SEs and T4SEs, providing a promising supplementary tool for further secretome studies.

## Introduction

Protein secretion plays an important role in coordinating the interactions between bacteria and their surrounding environment. Through a variety of secretion systems, bacteria can release different types of proteins into the extracellular environment or even directly inject them into eukaryotic host cells (Galan and Waksman, 2018; McQuade and Stock, 2018). Since bacterial secreted proteins are commonly involved in important physiological activities of host cells, they have become a new research hotspot in recent years. To date, nine different types of secretion systems have been discovered from Gram-negative bacteria, which are named type I secretion system (T1SS) to type IX secretion system (T9SS), respectively (Lasica et al., 2017; Lauber et al., 2018). Within these secretion systems, T1SS, T2SS, and T5SS can transport enzymes and other proteins into the surrounding environment, while type III secretion system (T3SS), type IV secretion system (T4SS), and type VI secretion system (T6SS) can transfer various effector proteins into host cells directly. These secreted effectors released through the latter three secretion systems are generally referred to as type III secreted effectors (T3SEs), type IV secreted effectors (T4SEs), and type VI secreted effectors (T6SEs) (An et al., 2018), and they can exert the virulence of Gram-negative bacteria in a number of ways, severely disrupting the normal function of host cells (Kim, 2018). Therefore, an in-depth study of secreted effectors is highly desirable for understanding the pathogenesis of bacteria and developing novel anti-microbial agents.

Over the past decade, dozens of machine learning-based computational approaches have been proposed to identify different types of secreted effectors (Zeng and Zou, 2019), including support vector machine (SVM) (Samudrala et al., 2009; Yang et al., 2010; Wang et al., 2011, 2014, 2017; Dong et al., 2013; Zou et al., 2013; Goldberg et al., 2016; Esna Ashari et al., 2019a,b), random forest (RF) (Yang et al., 2013), artificial neural network (ANN) (Löwer and Schneider, 2009), naive Bayes (NB) (Arnold et al., 2009), hidden Markov model (HMM) (Xu et al., 2010; Lifshitz et al., 2013; Wang et al., 2013), logistic regression (LR) (Esna Ashari et al., 2018), decision tree (DT) (Wang et al., 2019a), gradient boosting (Chen et al., 2020), deep learning (DL) (Xue et al., 2018, 2019; Açıcı et al., 2019; Fu and Yang, 2019; Hong et al., 2020; Li et al., 2020a), and their ensemble methods (Burstein et al., 2009; Hobbs et al., 2016; Wang et al., 2018, 2019b; Xiong et al., 2018; Li et al., 2020b). Some of these methods have achieved relatively high predictive accuracy, while they can recognize only one type of secreted effector, such as SIEVE (Samudrala et al., 2009), EffectiveT3 (Arnold et al., 2009), T3_MM (Wang et al., 2013), GenSET (Hobbs et al., 2016), Bastion3 (Wang et al., 2019a), DeepT3 (Xue et al., 2019), WEDeepT3 (Fu and Yang, 2019), ACNNT3 (Li et al., 2020a), and EP3 (Li et al., 2020b) for T3SEs; T4EffPred (Zou et al., 2013), T4SEpre (Wang et al., 2014), DeepT4 (Xue et al., 2018), PredT4SE-Stack (Xiong et al., 2018), Bastion4 (Wang et al., 2019b), T4SE-XGB (Chen et al., 2020), and CNN-T4SE (Hong et al., 2020) for T4SEs; and Bastion6 (Wang et al., 2018) for T6SEs. It is important to note that due to the small number of T6SEs for model construction, researchers usually pay more attention to identifying T3SEs and T4SEs rather than T6SEs. In addition, several multi-label classifiers have been developed to identify different types of Gram-negative bacterial secreted proteins simultaneously (Yu et al., 2013; Ding and Zhang, 2016; Liang et al., 2018; Yu et al., 2018; Kong and Zhang, 2019), but they are not good at distinguishing between T3SEs and T4SEs. Both T3SEs and T4SEs are non-classical secreted proteins (without classical N-terminal signal peptides) (Liang and Zhang, 2018; Zhang et al., 2020), and some of them have similar evolutionary conserved profiles and sequence motifs (Zou et al., 2013), so it is difficult to distinguish them accurately using current methods.

In this paper, we explore the use of various DL architectures and feature descriptors to identify and classify T3SEs and T4SEs. Four different DL architectures are used to build the classification models, including the convolutional neural networks (CNNs), recurrent neural networks (RNNs), convolutional-RNNs (CNN-RNNs), and deep neural networks (DNNs). For the CNN, RNN, and CNN-RNN architectures, we first characterize protein sequences using dictionary encoding and then generate amino-acid character embedding vectors to learn the features of two types of secreted effectors. The DNN architecture is designed as a multilayered neural network, whose input layer is fed traditional features or descriptors, including amino acid composition (AAC), dipeptide composition (DC), position-specific scoring matrix (PSSM), and their different combinations. We carry out extensive experiments for comparison and present a systematic analysis. Our results show that a hybrid neural network (architectures: RNN + DNN; features: dictionary encoding + AAC + DC) performs better than other models on the test and independent test datasets, enabling accurate classification of T3SEs and T4SEs. We also achieve interpretable DL for T3SEs and T4SEs classification via an advanced dimensionality reduction procedure and visualization, which unravels the predictions of models. Based on these results, we develop a DL approach, which is called DeepT3_4, by implementing both the raw sequence and sequence-derived features of effector proteins into the hybrid model. DeepT3_4 helps to understand the similar sequences and structures for some of T3SEs and T4SEs, facilitating the refined studies of different types of secreted effectors.

## Materials and Methods

### Dataset Collection and Processing

Reliable data are the primary factor in establishing stable and effective predictors, and all experimental data used in this study were extracted from the Bacterial Secreted Effector Protein DataBase (SecretEPDB) (An et al., 2017). SecretEPDB provides a comprehensive annotation of the T3SEs, T4SEs, and T6SEs, including sequence, structure, and function annotations for these secreted effectors. A total of 1230 T3SEs, 731 T4SEs, and 181 T6SEs were collected in this database, and we selected all of the T3SE and T4SE samples as original data to construct the training and test datasets.

In order to avoid redundancy and homology bias, all effector proteins in the original data were aligned by CD-HIT (Huang et al., 2010) with a maximum sequence identity of 25%. After that, only 302 T3SEs and 375 T4SEs were kept. Subsequently, 70% of this dataset was randomly selected for building the benchmark dataset and the remaining 30% was used to establish the independent test set (Jiang et al., 2017). Finally, the benchmark dataset contained 211 T3SEs and 263 T4SEs, while the independent test set was consisted of 91 T3SEs and 112 T4SEs (Supplementary Table S1).

For further evaluating the performance of our method and comparing with other state-of-the-art approaches, other two independent test datasets were established by searching publicly available articles. The independent test dataset 2 contains 108 T3SEs and 30 T4SEs, which were extracted directly from Bastion3 (Wang et al., 2019a) and Bastion4 (Wang et al., 2019b), respectively. The independent test dataset 3 is composed of 35 T3SEs and 75 T4SEs, which were collected from the studies of Yang et al. (2013) and Wang et al. (2017), respectively. In addition, other 1319 proteins were randomly selected to detect the performance of our method for identifying non-T3SEs and non-T4SEs.

### Feature Extraction

#### Dictionary Encoding

Each amino acid in the protein sequence is represented by an ordinal number, in which each of the 20 basic amino acids is assigned a number from 1 to 20 (e.g., alanine is assigned a number of 1) (Veltri et al., 2018). Thus, each protein is represented by a one-letter code and transformed into an *L*-dimensional vector, where *L* is the length of the protein.

#### Amino Acid Composition (AAC) and Dipeptide Composition (DC)

For each protein sequence, a 20-dimensional vector {*d*_1_, *d*_2_, …, *d*_20_} and a 400-dimensional vector {*d*_1_, *d*_2_, …, *d*_400_} are used to represent the compositions of 20 common amino acids and all 400 possible amino acid pairs, respectively. The 20 elements in {*d*_1_, *d*_2_, …, *d*_20_} represent the occurrence frequencies of each amino acid with a protein. The 400 elements in {*d*_1_, *d*_2_, …, *d*_400_} represent the frequencies of dipeptides.

#### Position-Specific Scoring Matrix (PSSM)

The PSSM profiles contain the evolutionary information of a protein. Each element in PSSM indicates the substitution scores of the individual residue at that specific position in the multiple sequence alignment. To generate PSSM, each protein sequence in our training and test datasets was searched against the Swiss-Prot database using the PSI-BLAST (Altschul and Koonin, 1998) with three iterations and a cutoff *E*-value of 0.001. The generated PSSM from PSI-BLAST includes *L* × 20 elements, where *L* is the length of a protein. This original profile is further used to calculate the PSSM feature by averaging the columns in PSSM profile and then is scaled to [−1, 1]. Finally, PSSM generates a 20-dimensional feature vector by characterizing a mutation of the corresponding amino acid type during the evolution process.

### Deep Neural Networks

As the most popular machine learning algorithm, DL has been successfully applied to solve various problems, such as image recognition, speech recognition, language translation, and biological data analysis (Jurtz et al., 2017; Tang et al., 2019). There have been four common variations of DNNs, including the CNNs, the RNNs, the CNN-RNNs, and the DNNs. The CNNs have outstanding spatial information analysis capabilities and have been successfully applied in the prediction of secreted effectors (Xue et al., 2018, 2019; Açıcı et al., 2019), protein solubility (Khurana et al., 2018), and crystallization (Elbasir et al., 2019). Compared to CNNs, RNNs can handle sequential inputs effectively and recognize sequence motifs of varying length extraordinarily well, making them the preferred choice for machine translation, text generation, and image captioning (Esteva et al., 2019). In order to integrate the advantages of the CNNs and RNNs, the CNN-RNNs have been developed in recent years and applied to a variety of biological problems (Quang and Xie, 2016; Pan et al., 2018; Tayara and Chong, 2019). As a typical representative of feedforward neural network (FNN), DNNs are composed of multiple perceptrons of different layers and are therefore very suitable for solving non-linear problems and have been widely used in data classification and other fields (Kruse et al., 2013).

### Deep Learning Architectures

To accurately classify the proteins of Gram-negative bacteria into separate secretion classes, we used DNNs with four different architectures. For the first three network architectures, including CNNs, RNNs, and CNN-RNNs, we encode the primary sequence using a dictionary amino acid representation as input and output one score between 0 and 1, corresponding to the probability of an effector protein of interest being a T3SE or a T4SE. The fourth architecture DNN is a standard multilayer neural network. The DNN model takes AAC, PSSM, DC, and their different combinations as inputs to predict the probability scores of two types of secreted effectors. We describe the overview of different DL architectures below.

The CNN consists of an embedding layer, a convolutional layer, a pooling layer, a fully connected layer, and an output layer. The first embedding layer transforms the input into a 256-dimensional vector representation. This transformation can best be thought of as a one-dimensional signal (over sequence position) spanning all amino acid signal channels. The input sequence is 1500 amino acids long, a number that was chosen to fit out dataset’s longest sequence. If the length of the sequence exceeds 1500 amino acids, the excess will be ignored; otherwise, the “X” character (unknown residue) will be padded at the tail of the sequence to fit the 1500 length. The second convolutional layer has 250 filters, where the filter width is set to five. The convolutional layer is then followed by a max-pooling layer with a non-overlapping window of size 2 to halve the size of the input. Subsequently, a fully connected layer consisted of 650 neurons with a dropout ratio of 20% is chosen to receive the flattening results of the pooling layer (Bogard et al., 2019). All layers whose activation is not specified explicitly use rectified linear unit (ReLU) activations. Finally, the output layer employs the sigmoid activation function to provide the predicted probability score for the test sequence.

The RNN is made up of three types of layers: an embedding layer, a biLSTM layer, and an output layer. The bidirectional long short-term memory (biLSTM) is an enhanced version of general RNNs in which the scalar-valued hidden layer of RNNs is replaced by a biLSTM memory block. The biLSTM layer is a forward–backward structure along the input sequence consisting of two relatively separated RNN layers. We explored biLSTM layers with 32, 64, 128, and 256 neurons and from one to four layers deep. A biLSTM layer with 64 neurons and one layer of depth gave the best performance. Dropout of 20% is applied to biLSTM layer to prevent overfitting. The final output layer utilizes a sigmoid activation function to process the output of the biLSTM layer and gives a value (the probability score) for each protein sequence.

The CNN-RNN incorporates an embedding layer with embedding size 256 along with a 1D convolutional layer with filter size = 5. The max-pooling layer subsamples the 1D signal by a factor of two. The flattened pooling output is passed to a biLSTM layer of 64 hidden neurons, which finally connects to a sigmoid activation function that outputs the predicted probability score.

The DNN is constructed from three fully connected layers with decreasing sizes of features vectors (e.g., 400, 200, and 100 for the DC) to reduce feature dimensions toward convergence of model training. As an additional precaution, a dropout probability of 20% is used in each layer.

The same RNN and DNN architectures are used to construct the hybrid model. The RNN and DNN models are trained separately, and their last hidden layers are further concatenated and inputted into a sigmoid activation node. The RNN architecture consists of an embedding layer and a biLSTM layer. The biLSTM layer has 64 hidden units followed by a dropout rate of 20%. The DNN model has three fully connected layers with 420, 210, and 105 neurons, respectively.

### Performance Evaluation

For evaluation, we used standard performance quantification metrics such as Recall (Sensitivity), Precision (PRE), Accuracy (ACC), F-value, and Matthew’s correlation coefficient (MCC), which are defined as follows:

(1) $$\text{Re}\,c\,a\,l\,l=\frac{T\,P}{T\,P+F\,N}$$

(2) $$P\,R\,E=\frac{T\,P}{T\,P+F\,P}$$

(3) $$A\,C\,C=\frac{T\,P+T\,N}{T\,P+F\,P+T\,N+F\,N}$$

(4) $$F-\text{value}=2\times \frac{T\,P}{2\,T\,P+F\,P+F\,N}$$

(5) $$M\,C\,C=\frac{T\,P\times T\,N-F\,N\times F\,P}{\sqrt{\left(T\,P+F\,N\right)\times \left(T\,N+F\,P\right)\times \left(T\,P+F\,P\right)\times \left(T\,N+F\,N\right)}}$$

where TP, FP, TN, and FN stand for true positive, false positive, true negative, and false negative, respectively.

### Implementation

All DNNs were implemented by using autoBioSeqpy (Jing et al., 2020). The autoBioSeqpy is an easy-to-use DL tool for biological sequence classification. The main advantage of this tool is its simplicity. Users only need to prepare the input dataset. After that, data encoding, model development, training, evaluation, and figure generation workflows can be run through the command line interface, by which users can modify the parameters of the workflows easily. In addition, autoBioSeqpy is designed to separate the data encoding and model configuration into two relatively independent parts. The DL models can be built using python code (i.e., written in.py files) or json files (saved by Keras), so that the model can be flexibly adjusted according to user needs. Currently, the tool has been upgraded to version 2.0, which supports more complex network models and incorporates model visualization function. For example, layerUMAP is a portable command-line tool included in the autoBioSeqpy tool suite, written in python, that makes use of the uniform manifold approximation and projection (UMAP) for visual understanding of DL models (Melville, 2019).

We sampled a variety of hyperparameter sets for different DL models, including embedding dimension (32, 64, 128, and 256), dropout rate (10, 20, 30, and 40%), batch size (25, 50, 75, and 100), epoch number (20, 40, 60, and 80), learning rate (0.001, 0.005, 0.01, 0.05, and 0.1), convolution kernel size (3, 5, 7, 9, and 11), number of filters (50, 100, 150, 200, and 250), and number of neurons in BiLSTM (32, 64, 128, and 256). We took the sampled parameter set with best performance (mean *MCC* score) and varied each parameter individually while keeping the rest constant.

During the training process, we used binary_crossentropy as loss function of the network and it has been optimized using the Adam optimizer approach with a learning rate 0.001. We trained all models with 40 epochs and a batch size of 25. The weights of the parameters were updated within 40 epochs, and at the end of each epoch, the intermediate validation metric is calculated. After the training, the optimized parameters were evaluated by the predictions from the test dataset. All the training was conducted on a Windows 10 workstation with an NVIDIA GTX 1060 GPU with CUDA 10.2.95. To interpret the model, we visualized the decision map of model in two dimensions. We used the output of the last hidden layer of the model as the extracted output features, which were then projected into a 2D manifold via UMAP. Next, we used a two-color scheme to refer to T3SE and T4SE based on the extracted output features.

## Results

### Overview of the Deep Learning Models

**FIGURE 1:**
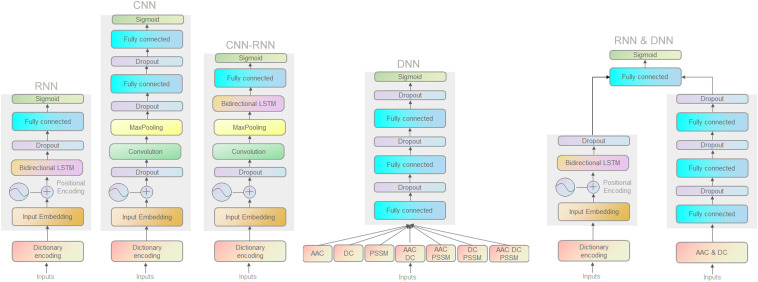
Schematic illustration of the deep neural network architectures.

We first used a DL classification tool (autoBioSeqpy) (Jing et al., 2020) to design and evaluate all 11 DL classifiers. Classifiers are divided into three categories: (1) different model architectures but with the same model inputs (CNN, RNN, and their combination CNN-RNN); (2) the same model architecture but with different model inputs (DNN); and (3) a hybrid architecture combining the above two categories (RNN and DNN). Figure 1 depicts all DL architectures in the effector classification. Details of the methods are reported in Section “Materials and Methods.”

### Effect of Model Architectures and Features on Performance

**FIGURE 2:**
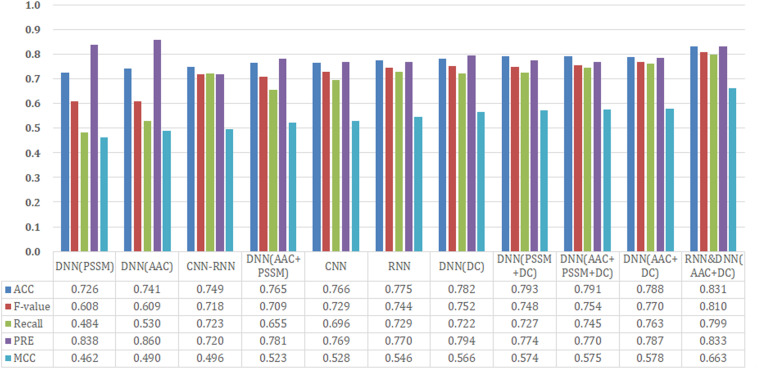
Performance comparison of different model architectures and features on the test dataset.

We analyzed the performance of 11 different models (CNN, RNN, CNN-RNN, seven DNN models with different input features, and a hybrid model) on our held-out test set. The benchmark dataset comprised 474 proteins, 70% of which was randomly extracted for establishing the training set, 20% for the test set, and the remaining 10% for the validation set. We performed an extensive random hyperparameter search for each model on the validation set, and then the top-performing tuned models were evaluated on the test set. A summary of our results is provided in Figure 2.

Since CNN, RNN, and CNN-RNN have the same model inputs, we first compared these three DL architectures. The RNN model afforded the best training performance with the highest scores of *Recall* (72.9%), *PRE* (77.0%), *ACC* (77.5%), *MCC* (0.546), and *F*-value (74.4%). The CNN model followed with an *ACC* of 76.6% and an *MCC* of 0.528. The CNN-RNN model showed the lowest performance (*ACC* = 74.9% and *MCC* = 0.496), which were lower than those of the RNN model as 2.6% and 0.050 on *ACC* and *MCC*, respectively.

Based on AAC, DC, PSSM, and their different combinations, seven different features were employed to build the DNN models. From Figure 2, it can be seen that DNN models trained by the single feature group (only AAC, PSSM, or DC) tended to obtain the relatively poor results, whereas those trained by a combination of features (AAC+PSSM, AAC+DC, PSSM+DC, and AAC+PSSM+DC) seemed to achieve better performance. For the seven DNN models, the model with AAC+DC yielded the best performance, and gave the highest scores of *Recall* (76.3%), *F*-value (77.0%), and *MCC* (0.578). The PSSM+DC model offered the highest *ACC* score (79.3%), but other four parameters were lower than the AAC+DC model. Although the model with AAC+PSSM+DC learns the most information, its overall predictive performance was also weaker than that of the model with AAC+DC. Surprisingly, the comprehensive performance of the model with single feature DC was almost comparable to those models trained with the combined features. This result indicates that DC is a very important feature for making a distinction between T3SEs and T4SEs.

**FIGURE 3:**
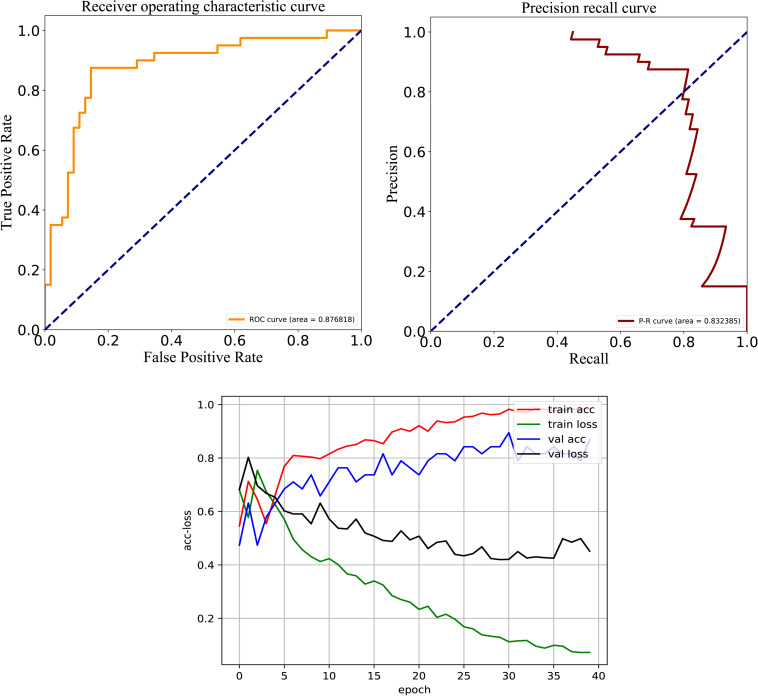
ROC, PR, and acc-loss curves generated by autoBioSeqpy tool for the hybrid deep learning model on the test dataset.

After careful analysis of above results, we proposed a hybrid model to integrate the advantages of the RNN and best DNN models. This hybrid DNN model yielded the best overall prediction performance for the test dataset, and provided the highest scores of *Recall* (77.9%), *PRE* (83.3%), *ACC* (83.1%), *MCC* (0.663), and *F*-value (81.0%). Therefore, we chose this hybrid model as the final prediction model for this study. The receiver operating characteristic (ROC) curve, precision recall (P-R) curve, and accuracy-loss (acc-loss) curve were exploited to evaluate the performance of the hybrid model (Figure 3). Area values under the ROC curve (auROC) and P-R curve (auPRC) for the hybrid model were 0.877 and 0.832, respectively. We also trained the RNN and DNN (AAC+DC) models separately to evaluate the robustness of the models, showing auROCs of (0.804 and 0.847) and auPRCs of (0.795 and 0.794), respectively (Supplementary Figures S1, S2). The results suggest that the RNN and DNN (AAC+DC) models learned different sets of features that complement each other for the task of distinguishing between two types of secreted effectors.

### Visualizing and Understanding Deep Learning Models

**FIGURE 4:**
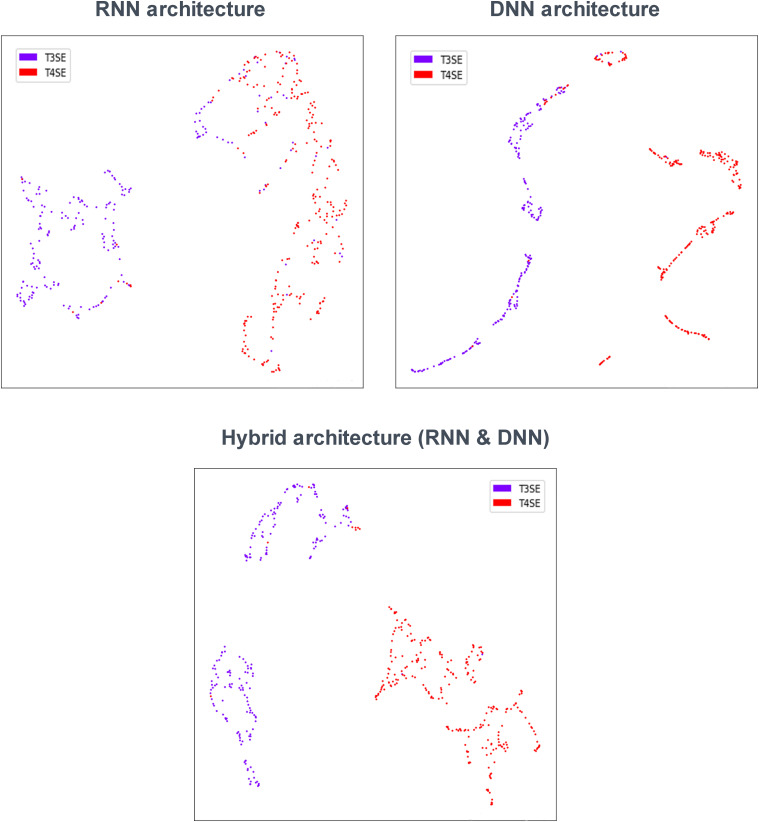
UMAP visualization of learned features.

To investigate the ability of DL models to distinguish two types of secreted effectors, we analyzed the features extracted from the last hidden layer of three classification models [RNN, DNN (AAC+DC), and RNN and DNN (AAC+DC)]. Figure 4 shows a UMAP (McInnes and Healy, 2018) for dimension reduction projection of these features. The points are color-coded based on the true class label. Therefore, T3SEs and T4SEs are characterized by purple and red points, respectively, with each point representing an effector. As shown in Figure 4, the features clearly distinguish the different secreted effectors. In the RNN architecture, some T3SEs are distributed across the T4SE cluster with no obvious pattern. The DNN and hybrid architectures have the advantage of very clearly clusters, which is consistent with the above classification results. Furthermore, studies have confirmed that T3SS could be divided into two subgroups, including the injectisome (non-flagellar) system and the fagellar system (Blocker et al., 2003; Puhar and Sansonetti, 2014). Therefore, as the secretory products of the T3SS, T3SEs could also be classified into two subtypes, which are shown by the two sub-populations in Figure 4. Thus, this result implies that T3SEs do have different sequences and conserved patterns as well.

### Performance of Different Model Architectures and Features on the Independent Test Set

**FIGURE 5:**
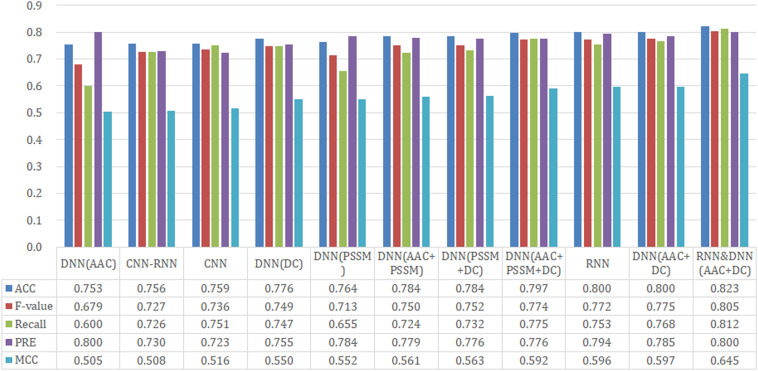
Performance comparison of different model architectures and features on the independent test set.

**FIGURE 6:**
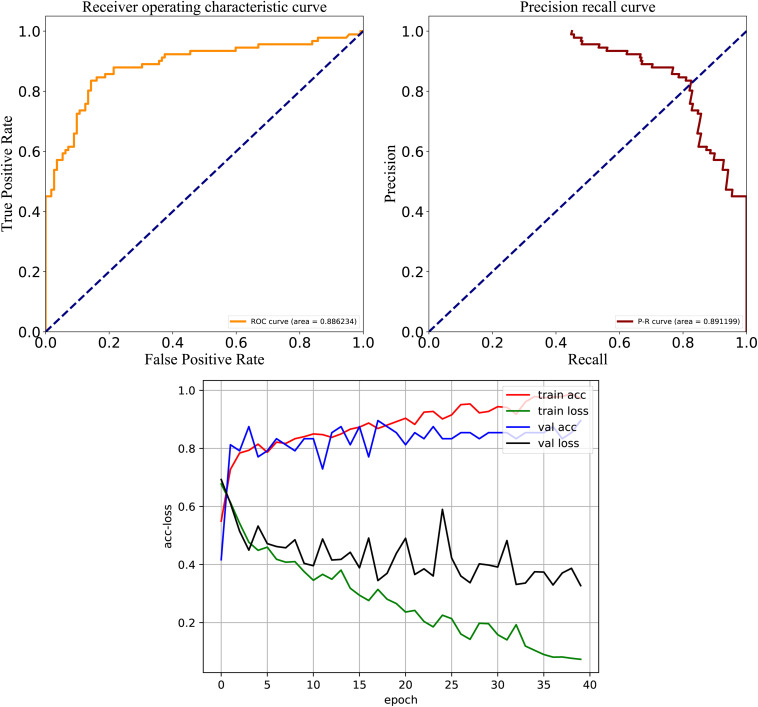
ROC, PR, and acc-loss curves generated by autoBioSeqpy tool for the hybrid deep learning model on the independent test set.

To test model performance on external data, an independent test set was obtained whose data were never used for training and testing. We used this dataset to further compare the predictive performance of models established by different architectures and features, and the results are shown in Figure 5. For the three DL architectures whose inputs are dictionary-encoded sequences, the RNN model also yielded the best overall prediction performance, achieving the highest scores of *Recall* (75.3%), *PRE* (79.4%), *ACC* (80.0%), *MCC* (0.596), and *F*-value (77.2%). The CNN-RNN model got the worst predictive performance, including the lowest scores of *Recall* (72.6%), *ACC* (75.6%), *MCC* (0.508), and *F*-value (72.7%). The AAC+DC model also afforded the best overall predictive performance among the seven DNN models, receiving the highest scores for *ACC* (80.0%), *MCC* (0.597), and *F*-value (77.5%). Finally, the hybrid RNN and DNN (AAC+DC) model obtained the best overall predictive performance on the independent test set, providing the highest scores for *Recall* (81.2%), *PRE* (80.0%), *ACC* (82.3%), *MCC* (0.645), and *F*-value (80.5%), respectively. We then evaluated the performance of RNN, DNN (AAC+DC), and their hybrid model using ROC, PR, and acc-loss curves (Supplementary Figures S3, S4 and Figure 6). In terms of auROC and auPRC, the hybrid model also performed better than other two models. These results further suggest that combining learned features of RNN and DNN models can deliver a better model compared with individual models.

### Development of DeepT3_4 and Comparison With Other Existing Methods

To further evaluate the performance of our hybrid DL model (named DeepT3_4), we used other two independent test datasets to compare the performance of DeepT3_4 with other three state-of-the-art approaches, including a typical T3SE predictor-Bastion3 (Wang et al., 2019a) and two representative T4SE classifiers-Bastion4 (Wang et al., 2019b) and CNN-T4SE (Hong et al., 2020). For the independent test dataset 2, all prediction results are listed in Table 1. As shown in the table, Bastion3 correctly identified all 108 T3SEs, but 12 T4SEs were incorrectly predicted as T3SEs; 29 T4SEs were correctly identified by Bastion4, but 25 T3SEs were incorrectly predicted as T4SEs. CNN-T4SE correctly identified the maximum number of T4SEs (29), but got the minimum number of T3SEs (59). When using DeepT3_4, 101 T3SEs and 26 T4SEs were correctly identified, and seven T3SEs and four T4SEs were misclassified. Although DeepT3_4 did not obtain the highest *Recall* for T3SEs and the highest *PRE* for T4SEs, it yielded the best overall prediction performance here. DeepT3_4 gave the highest scores of *ACC* (92.0%), *MCC* (0.775), and *F*-value (94.8%), which provided a 0.7%, 0.1%, and 0.040 improvement in *ACC*, *F*-value, and *MCC*, respectively. These results indicate that DeepT3_4 is stable and reliable in distinguishing T3SEs and T4SEs.

**TABLE 1:** Performance Comparisons of DeepT3_4 with other three methods on the independent test dataset 2.

**Model**	**TP**	**TN**	**FN**	**FP**	**ACC**	***F*-value**	**Recall**	**PRE**	**MCC**
					**(%)**	**(%)**	**(%)**	**(%)**	
Bastion3	108	18	0	12	91.3	94.7	100.0	90.0	0.735
Bastion4	83	29	25	1	81.2	86.5	76.9	98.8	0.621
CNN-T4SE	59	29	49	1	63.8	70.2	54.6	98.3	0.427
DeepT3_4	101	26	7	4	92.0	94.8	93.5	96.2	0.775

For the independent test dataset 3, all results are shown in Table 2. As we can see from this table, Bastion3 acquired the best overall prediction performance with the highest scores of *ACC* (95.5%), *F*-value (93.0%), *Recall* (94.3%), and *MCC* (0.896). The performance of DeepT3_4 is slightly lower than that of Bastion3, and afforded the second highest scores of *ACC* (94.5%), *F*-value (91.4%), *Recall* (91.4%), and *MCC* (0.874). Though Bastion4 and CNN-T4SE got the highest score of *PRE* (100.0%), their overall prediction performances were worse than those of Bastion3 and DeepT3_4. It is noteworthy that for most of query sequences (known secreted effectors) in the independent test dataset 3, Bastion3 and Bastion4 did not provide the prediction results, but directly gave the search results of BastionDB and all results were marked as *Exp*. If both of Bastion3 and Bastion4 give the prediction results for all query sequences, we believe that DeepT3_4 will perform better than them.

**TABLE 2:** Performance Comparisons of DeepT3_4 with other three methods on the independent test dataset 3.

**Model**	**TP**	**TN**	**FN**	**FP**	**ACC**	***F*-value**	**Recall**	**PRE**	**MCC**
					**(%)**	**(%)**	**(%)**	**(%)**	
Bastion3	33	72	2	3	95.5	93.0	94.3	91.7	0.896
Bastion4	27	75	8	0	92.7	87.1	77.1	100.0	0.835
CNN-T4SE	28	75	7	0	93.6	88.9	80.0	100.0	0.855
DeepT3_4	32	72	3	3	94.5	91.4	91.4	91.4	0.874

### Model Robustness Evaluation

To assess the effect of data scale on the predictive performance of DeepT3_4, we calculated and plotted learning curves to observe the relationship between the performance and data size. To generate learning curves, an external resampling mechanism with replacement was used to generate subsets with five different scales: 20, 40, 60, 80, and 100%. After resampling, the subset was split into five training-test groups for cross-validation. Each resampling was repeated 10 times to measure the robustness of the DL model. Thus, a total of 250 models (10 replicates ^∗^ five scales ^∗^ five folds) were built for predicting the generated test sets. Supplementary Figures S5, S6 show the learning curves of the DeepT3_4 model using the ACC and MCC metrics. The DeepT3_4 model becomes relatively stable when the scale of the dataset reaches 60% (about 325 samples). On this scale, the ACC and MCC scores in cross-validation are 81.0 ± 3.0% and 0.620 ± 0.060, respectively. Except for the learning curve, we also used fivefold cross-validation on a 100% scale dataset to further evaluate the generalizability of the model. The detailed results are shown in Supplementary Table S2. The DeepT3_4 achieves the average scores of 83.9 ± 2.6% for ACC and 0.677 ± 0.052 for MCC, which is consistent with the results of 10-time test (Figure 2). All together, these results illustrate the robustness of DeepT3_4, even on the small sample dataset.

## Discussion

In recent years, many excellent works have been done in the field of secreted effector prediction, such as Bastion3 (Wang et al., 2019a) and DeepT3 (Xue et al., 2019) for T3SEs and Bastion4 (Wang et al., 2019b) and CNN-T4SE (Hong et al., 2020) for T4SEs. Different from these studies, we developed a hybrid DL approach by integrating RNN and DNN architectures to classify T3SEs and T4SEs in this work. We have carried out extensive experiments for comparison and have presented an in-depth analysis. For both the benchmark and independent test sets, the hybrid DNN model shows a consistently better performance than the others. The innovations of this study are as follows: (i) to the best of our knowledge, this is the first study to use DL to classify T3SEs and T4SEs; (ii) different DL architectures and features are employed to construct the predictors; (iii) clustering and visualization of model-extracted features using UMAP; (iv) the experimental results confirm that some of T3SEs and T4SEs may have similar evolutionary conservatism profiles and sequence motifs, which leads to limitations in the classification performance of computational methods.

The secretion signal of T3SEs is generally located at the N-terminal sequences (Yang et al., 2013), while the secretion signal of T4SEs is commonly found in the C-terminal sequences (Nagai et al., 2005). Therefore, some state-of-the-art methods choose only 50–100 N-terminal amino acid residues to identify T3SEs (Wang et al., 2013; Yang et al., 2013; Xue et al., 2019), or only 100 C-terminal amino acid residues to predict T4SEs (Zou et al., 2013; Wang et al., 2014; Xue et al., 2018). In order to assess the role of N-terminal or C-terminal sequence features in the classification of T3SEs and T4SEs, we calculated the sequence-based features within the first 100 N-terminal residues, the last 100 C-terminal residues, and the whole protein sequences using the best hybrid model, and further compared their performance using the independent test set consisting of 91 T3SEs and 112 T4SEs. All test results are listed in Supplementary Table S3. As can be seen from the table, the hybrid model trained by the full protein sequences achieved the best overall prediction performance and afforded the highest scores of *Recall* (81.2%), *PRE* (80.0%), *ACC* (82.3%), *MCC* (0.645), and *F*-value (80.5%). However, the performance of the hybrid models trained on the first 100 N-terminal and last 100 C-terminal residues is lower than that of the full-length sequence. These results suggest that the full sequences can better characterize the two types of secreted effectors.

We further developed DeepT3_4 to be able to predict non-T3SEs and non-T4SEs. To further estimate the performance of DeepT3_4, we employed a new dataset for a ternary classification, which is composed of 1319 other proteins. When tested on the new test set 10 times, DeepT3_4 obtained an overall average *ACC* of 88.2%, which is higher than that of the binary classification (*ACC* = 82.3%), suggesting that the addition of other types of protein sequences does not affect the predictive performance of our method.

In order to gain insight into the pathogenesis of bacteria and to effectively develop new drugs, an increasing number of studies have been conducted on various secreted effectors. Although DeepT3_4 can distinguish between T3SEs and T4SEs, there is still some room for further improvement. Moreover, there are still many issues to be solved in the study of secreted effectors. For example, T6SEs are widespread in various Gram-negative bacteria, but only a few computational methods are currently available to accurately identify them (Wang et al., 2018, 2020; Sen et al., 2019). The T3SS and T4SS can be divided into different subgroups (Costa et al., 2015), and thus their secretory products, T3SEs and T4SEs are also classified into different subfamilies (Bi et al., 2013; Zou et al., 2013). However, more detailed studies of the subfamilies of T3SEs and T4SEs are still rare. In addition, a new predictor has been built to recognize potential non-classical secreted proteins of Gram-positive bacteria recently (Zhang et al., 2020), which may spark a wave of researches on bacterial non-classical secreted proteins. Overall, we propose an effective computational method to accurately differentiate between T3SEs and T4SEs in this work, and hope it could facilitate more relevant researches on bacterial secreted effectors.

## Data Availability Statement

Publicly available datasets were analyzed in this study. These data can be found here: https://github.com/jingry/autoBioSeqpy/tree/2.0/examples/T3T4.

## Author Contributions

JL and LY conceived the study and wrote the manuscript. RJ contributed to the design, implementation, and testing of the model. LY and FL performed the data analysis. All authors read and agreed to the published version of the manuscript.

## Conflict of Interest

The authors declare that the research was conducted in the absence of any commercial or financial relationships that could be construed as a potential conflict of interest.

## References

[B1] Açıcı K. Aşuroğlu T. Erdaş ÇB. Oğul H. (2019). T4SS effector protein prediction with deep learning. *Data* 4:45 10.3390/data4010045

[B2] Altschul S. F. Koonin E. V. (1998). Iterated Profile Searches with PSI-BLAST—A tool for discovery in protein databases. *Trends Biochem. Sci.* 23 444–447. 10.1016/S0968-0004(98)01298-5 9852764

[B3] An Y. Wang J. Li C. Leier A. Marquez-Lago T. Wilksch J. (2018). Comprehensive assessment and performance improvement of effector protein predictors for bacterial secretion systems III, IV and VI. *Brief. Bioinform.* 19 148–161. 10.1093/bib/bbw100 27777222

[B4] An Y. Wang J. Li C. Revote J. Zhang Y. Naderer T. (2017). SecretEPDB: a comprehensive web-based resource for secreted effector proteins of the bacterial types III, IV and VI secretion systems. *Sci. Rep.* 7:41031. 10.1038/srep41031 28112271 PMC5253721

[B5] Arnold R. Brandmaier S. Kleine F. Tischler P. Heinz E. Behrens S. (2009). Sequence-based prediction of type III secreted proteins. *PLoS Pathog.* 5:e1000376. 10.1371/journal.ppat.1000376 19390696 PMC2669295

[B6] Bi D. Liu L. Tai C. Deng Z. Rajakumar K. Ou H. Y. (2013). SecReT4: a web-based bacterial type IV secretion system resource. *Nucleic Acids Res.* 41 D660–D665. 10.1093/nar/gks1248 23193298 PMC3531058

[B7] Blocker A. Komoriya K. Aizawa S. (2003). Type III secretion systems and bacterial flagella: insights into their function from structural similarities. *Proc. Natl. Acad. Sci. U. S. A.* 100 3027–3030. 10.1073/pnas.0535335100 12631703 PMC152238

[B8] Bogard N. Linder J. Rosenberg A. B. Seelig G. (2019). A deep neural network for predicting and engineering alternative polyadenylation. *Cell* 178 91–106. 10.1016/j.cell.2019.04.046 31178116 PMC6599575

[B9] Burstein D. Zusman T. Degtyar E. Viner R. Segal G. Pupko T. (2009). Genome-scale identification of *Legionella pneumophila* effectors using a machine learning approach. *PLoS Pathog.* 5:e1000508. 10.1371/journal.ppat.1000508 19593377 PMC2701608

[B10] Chen T. Wang X. Chu Y. Wei D. Xiong Y. (2020). T4SE-XGB: interpretable sequence-based prediction of type IV secreted effectors using eXtreme gradient boosting algorithm. *bioRxiv [Preprint]* 10.1101/2020.06.18.158253 PMC7541839 33072049

[B11] Costa T. R. Felisberto-Rodrigues C. Meir A. Prevost M. S. Redzej A. Trokter M. (2015). Secretion systems in Gram-negative bacteria: structural and mechanistic insights. *Nat. Rev. Microbiol.* 13 343–359. 10.1038/nrmicro3456 25978706

[B12] Ding S. Zhang S. (2016). A Gram-negative bacterial secreted protein types prediction method based on PSI-BLAST profile. *Biomed Res. Int*. 2016:3206741. 10.1155/2016/3206741 27563663 PMC4985605

[B13] Dong X. Zhang Y. J. Zhang Z. (2013). Using weakly conserved motifs hidden in secretion signals to identify type-III effectors from bacterial pathogen genomes. *PLoS One* 8:e56632. 10.1371/journal.pone.0056632 23437191 PMC3577856

[B14] Elbasir A. Moovarkumudalvan B. Kunji K. Kolatkar P. R. Mall R. Bensmail H. (2019). DeepCrystal: a deep learning framework for sequence-based protein crystallization prediction. *Bioinformatics* 35 2216–2225. 10.1093/bioinformatics/bty953 30462171

[B15] Esna Ashari Z. Brayton K. A. Broschat S. L. (2019a). Prediction of T4SS effector proteins for Anaplasma phagocytophilum Using OPT4e, a new software tool. *Front. Microbiol.* 10:1391. 10.3389/fmicb.2019.01391 31293540 PMC6598457

[B16] Esna Ashari Z. Brayton K. A. Broschat S. L. (2019b). Using an optimal set of features with a machine learning-based approach to predict effector proteins for *Legionella pneumophila*. *PLoS One* 14:e0202312 10.1101/383570 PMC6347213 30682021

[B17] Esna Ashari Z. Dasgupta N. Brayton K. A. Broschat S. L. (2018). An optimal set of features for predicting type IV secretion system effector proteins for a subset of species based on a multi-level feature selection approach. *PLoS One* 13:e0197041. 10.1371/journal.pone.0197041 29742157 PMC5942808

[B18] Esteva A. Robicquet A. Ramsundar B. Kuleshov V. DePristo M. Chou K. (2019). A guide to deep learning in healthcare. *Nat. Med.* 25 24–29. 10.1038/s41591-018-0316-z 30617335

[B19] Fu X. Yang Y. (2019). WEDeepT3: predicting type III secreted effectors based on word embedding and deep learning. *Quant. Biol.* 7 293–301. 10.1007/s40484-019-0184-7

[B20] Galan J. E. Waksman G. (2018). Protein-Injection machines in bacteria. *Cell* 172 1306–1318. 10.1016/j.cell.2018.01.034 29522749 PMC5849082

[B21] Goldberg T. Rost B. Bromberg Y. (2016). Computational prediction shines light on type III secretion origins. *Sci. Rep.* 6:34516. 10.1038/srep34516 27713481 PMC5054392

[B22] Hobbs C. K. Porter V. L. Stow M. L. Siame B. A. Tsang H. H. Leung K. Y. (2016). Computational approach to predict species-specific type III secretion system (T3SS) effectors using single and multiple genomes. *BMC Genomics* 17:1048. 10.1186/s12864-016-3363-1 27993130 PMC5168842

[B23] Hong J. Luo Y. Mou M. Fu J. Zhang Y. Xue W. (2020). Convolutional neural network-based annotation of bacterial type IV secretion system effectors with enhanced accuracy and reduced false discovery. *Brief. Bioinform.* 21 1825–1836. 10.1093/bib/bbz120 31860715

[B24] Huang Y. Niu B. Gao Y. Fu L. Li W. (2010). CD-HIT Suite: a web server for clustering and comparing biological sequences. *Bioinformatics* 26 680–682. 10.1093/bioinformatics/btq003 20053844 PMC2828112

[B25] Jiang J. Chen Y. Narayan A. (2017). Offline-enhanced reduced basis method through adaptive construction of the surrogate training set. *J. Sci. Comput.* 73 853–875. 10.1007/s10915-017-0551-3

[B26] Jing R. Li Y. Xue L. Liu F. Li M. Luo J. (2020). autoBioSeqpy: a deep learning tool for the classification of biological sequences. *J. Chem. Inf. Model*. 60 3755–3764. 10.1021/acs.jcim.0c00409 32786512

[B27] Jurtz V. I. Johansen A. R. Nielsen M. Armenteros J. J. A. Nielsen H. Sønderby C. K. (2017). An introduction to deep learning on biological sequence data: examples and solutions. *Bioinformatics* 33 3685–3690. 10.1093/bioinformatics/btx531 28961695 PMC5870575

[B28] Khurana S. Rawi R. Kunji K. Chuang G. Y. Bensmail H. Mall R. (2018). DeepSol: a deep learning framework for sequence-based protein solubility prediction. *Bioinformatics* 34 2605–2613. 10.1093/bioinformatics/bty166 29554211 PMC6355112

[B29] Kim B. S. (2018). The modes of action of MARTX toxin effector domains. *Toxins* 10:507. 10.3390/toxins10120507 30513802 PMC6315884

[B30] Kong L. Zhang L. (2019). An ensemble method for multi-type Gram negative bacterial secreted protein prediction by integrating different PSSM-based features. *SAR QSAR Environ. Res.* 30 181–194. 10.1080/1062936X.2019.1573438 30739484

[B31] Kruse R. Borgelt C. Klawonn F. Moewes C. Steinbrecher M. Held P. (2013). “Multi-layer perceptrons,” in *Computational Intelligence*, ed. Inkpen D. (Berlin: Springer), 47–81.

[B32] Lasica A. M. Ksiazek M. Madej M. Potempa J. (2017). The type IX secretion system (T9SS): highlights and recent insights into its structure and function. *Front. Cell. Infect. Microbiol.* 7:215. 10.3389/fcimb.2017.00215 28603700 PMC5445135

[B33] Lauber F. Deme J. C. Lea S. M. Berks B. C. (2018). Type 9 secretion system structures reveal a new protein transport mechanism. *Nature* 564 77–82. 10.1038/s41586-018-0693-y 30405243 PMC6927815

[B34] Li J. Li Z. Luo J. Yao Y. (2020a). ACNNT3: Attention-CNN Framework for prediction of sequence-based bacterial type III secreted effectors. *Comput. Math. Methods Med.* 2020:3974598. 10.1155/2020/3974598 32328150 PMC7157791

[B35] Li J. Wei L. Guo F. Zou Q. (2020b). EP3: an ensemble predictor that accurately identifies type III secreted effectors. *Brief. Bioinform* bbaa008. 10.1093/bib/bbaa008 32043137

[B36] Liang Y. Zhang S. (2018). Identify Gram-negative bacterial secreted protein types by incorporating different modes of PSSM into Chou’s general PseAAC via Kullback-Leibler divergence. *J. Theor. Biol.* 454 22–29. 10.1016/j.jtbi.2018.05.035 29857085

[B37] Liang Y. Zhang S. Ding S. (2018). Accurate prediction of Gram-negative bacterial secreted protein types by fusing multiple statistical features from PSI-BLAST profile. *SAR QSAR Environ. Res.* 29 469–481. 10.1080/1062936X.2018.1459835 29688029

[B38] Lifshitz Z. Burstein D. Peeri M. Zusman T. Schwartz K. Shuman H. A. (2013). Computational modeling and experimental validation of the *Legionella* and *Coxiella* virulence-related type-IVB secretion signal. *Proc. Natl. Acad. Sci. U.S.A.* 110 E707–E715. 10.1073/pnas.1215278110 23382224 PMC3581968

[B39] Löwer M. Schneider G. (2009). Prediction of type III secretion signals in genomes of Gram-negative bacteria. *PLoS One* 4:e5917. 10.1371/journal.pone.0005917 19526054 PMC2690842

[B40] McInnes L. Healy J. (2018). UMAP: uniform manifold approximation and projection for dimension reduction. *ArXiv [Preprint]* 10.21105/joss.00861

[B41] McQuade R. Stock S. P. (2018). Secretion systems and secreted proteins in Gram-negative entomopathogenic bacteria: their roles in insect virulence and beyond. *Insects* 9:68. 10.3390/insects9020068 29921761 PMC6023292

[B42] Melville J. (2019). *uwot**: The Uniform Manifold Approximation and Projection (UMAP) Method for Dimensionality Reduction.* Available online at: https://github.com/jlmelville/uwot (accessed October, 2020).

[B43] Nagai H. Cambronne E. D. Kagan J. C. Amor J. C. Kahn R. A. Roy C. R. (2005). A C-terminal translocation signal required for Dot/Icm-dependent delivery of the *Legionella* RalF protein to host cells. *Proc. Natl. Acad. Sci. U. S. A.* 102 826–831. 10.1073/pnas.0406239101 15613486 PMC545534

[B44] Pan X. Rijnbeek P. Yan J. Shen H. B. (2018). Prediction of RNA-protein sequence and structure binding preferences using deep convolutional and recurrent neural networks. *BMC Genomics* 19:511. 10.1186/s12864-018-4889-1 29970003 PMC6029131

[B45] Puhar A. Sansonetti P. J. (2014). Type III secretion system. *Curr. Biol*. 24 784–791. 10.1016/j.cub.2014.07.016 25202865

[B46] Quang D. Xie X. (2016). DanQ: a hybrid convolutional and recurrent deep neural network for quantifying the function of DNA sequences. *Nucleic Acids Res.* 44:e107. 10.1093/nar/gkw226 27084946 PMC4914104

[B47] Samudrala R. Heffron F. McDermott J. E. (2009). Accurate prediction of secreted substrates and identification of a conserved putative secretion signal for type III secretion systems. *PLoS Pathog.* 5:e1000375. 10.1371/journal.ppat.1000375 19390620 PMC2668754

[B48] Sen R. Nayak L. De R. K. (2019). PyPredT6: a python-based prediction tool for identification of Type VI effector proteins. *J. Bioinf. Comput. Biol.* 17:1950019. 10.1142/S0219720019500197 31288641

[B49] Tang B. Pan Z. Yin K. Khateeb A. (2019). Recent advances of deep learning in bioinformatics and computational biology. *Front. Genet.* 10:214. 10.3389/fgene.2019.00214 30972100 PMC6443823

[B50] Tayara H. Chong K. T. (2019). Improving the quantification of DNA sequences using evolutionary information based on deep learning. *Cells* 8:1635. 10.3390/cells8121635 31847308 PMC6952993

[B51] Veltri D. Kamath U. Shehu A. (2018). Deep learning improves antimicrobial peptide recognition. *Bioinformatics* 34 2740–2747. 10.1093/bioinformatics/bty179 29590297 PMC6084614

[B52] Wang C. Li J. Zhang Y. Guo M. (2020). Identification of Type VI effector proteins using a novel ensemble classifier. *IEEE Access* 8 75085–75093. 10.1109/ACCESS.2020.2985111

[B53] Wang J. Li J. Yang B. Xie R. Marquez-Lago T. T. Leier A. (2019a). Bastion3: a two-layer ensemble predictor of type III secreted effectors. *Bioinformatics* 35 2017–2028. 10.1093/bioinformatics/bty914 30388198 PMC7963071

[B54] Wang J. Yang B. An Y. Marquez-Lago T. T. Leier A. Wilksch J. (2019b). Systematic analysis and prediction of type IV secreted effector proteins by machine learning approaches. *Brief. Bioinform.* 20 931–951. 10.1093/bib/bbx164 29186295 PMC6585386

[B55] Wang J. Yang B. Leier A. Marquez-Lago T. T. Hayashida M. Rocker A. (2018). Bastion6: a bioinformatics approach for accurate prediction of type VI secreted effectors. *Bioinformatics* 34 2546–2555. 10.1093/bioinformatics/bty155 29547915 PMC6061801

[B56] Wang Y. Guo Y. Pu X. Li M. (2017). Effective prediction of bacterial type IV secreted effectors by combined features of both C-termini and N-termini. *J. Comput. Aided Mol. Des.* 31 1029–1038. 10.1007/s10822-017-0080-z 29127583

[B57] Wang Y. Sun M. Bao H. White A. P. (2013). T3_MM: a Markov model effectively classifies bacterial type III secretion signals. *PLoS One* 8:e58173. 10.1371/journal.pone.0058173 23472154 PMC3589343

[B58] Wang Y. Wei X. Bao H. Liu S. L. (2014). Prediction of bacterial type IV secreted effectors by C-terminal features. *BMC Genomics* 15:50. 10.1186/1471-2164-15-50 24447430 PMC3915618

[B59] Wang Y. Zhang Q. Sun M. A. Guo D. (2011). High-accuracy prediction of bacterial type III secreted effectors based on position-specific amino acid composition profiles. *Bioinformatics* 27 777–784. 10.1093/bioinformatics/btr021 21233168

[B60] Xiong Y. Wang Q. Yang J. Zhu X. Wei D. Q. (2018). PredT4SE-Stack: prediction of bacterial type IV secreted effectors from protein sequences using a stacked ensemble method. *Front. Microbiol.* 9:2571. 10.3389/fmicb.2018.02571 30416498 PMC6212463

[B61] Xu S. Zhang C. Miao Y. Gao J. Xu D. (2010). Effector prediction in host-pathogen interaction based on a Markov model of a ubiquitous EPIYA motif. *BMC Genomics* 11:S1. 10.1186/1471-2164-11-S3-S1 21143776 PMC2999339

[B62] Xue L. Tang B. Chen W. Luo J. (2018). A deep learning framework for sequence-based bacteria type IV secreted effectors prediction. *Chemom. Intell. Lab. Syst.* 183 134–139. 10.1016/j.chemolab.2018.11.002

[B63] Xue L. Tang B. Chen W. Luo J. (2019). DeepT3: deep convolutional neural networks accurately identify Gram-negative bacterial type III secreted effectors using the N-terminal sequence. *Bioinformatics* 35 2051–2057. 10.1093/bioinformatics/bty931 30407530

[B64] Yang X. Guo Y. Luo J. Pu X. Li M. (2013). Effective identification of Gram-negative bacterial type III secreted effectors using position-specific residue conservation profiles. *PLoS One* 8:e84439. 10.1371/journal.pone.0084439 24391954 PMC3877298

[B65] Yang Y. Zhao J. Morgan R. L. Ma W. Jiang T. (2010). Computational prediction of type III secreted proteins from Gram-negative bacteria. *BMC Bioinformatics* 11(Suppl. 1):S47. 10.1186/1471-2105-11-S1-S47 20122221 PMC3009519

[B66] Yu L. Liu F. Du L. Li Y. (2018). An improved approach for rapidly identifying different types of Gram-negative bacterial secreted proteins. *Nat. Sci.* 10 168–177. 10.4236/ns.2018.105018

[B67] Yu L. Luo J. Guo Y. Li Y. Pu X. Li M. (2013). In silico identification of Gram-negative bacterial secreted proteins from primary sequence. *Comput. Biol. Med.* 43 1177–1181. 10.1016/j.compbiomed.2013.06.001 23930811

[B68] Zeng C. Zou L. (2019). An account of *in silico* identification tools of secreted effector proteins in bacteria and future challenges. *Brief. Bioinform.* 20 110–129. 10.1093/bib/bbx078 28981574

[B69] Zhang Y. Yu S. Xie R. Li J. Leier A. Marquez-Lago T. T. (2020). PeNGaRoo, a combined gradient boosting and ensemble learning framework for predicting non-classical secreted proteins. *Bioinformatics* 36 704–712. 10.1093/bioinformatics/btz629 31393553

[B70] Zou L. Nan C. Hu F. (2013). Accurate prediction of bacterial type IV secreted effectors using amino acid composition and PSSM profiles. *Bioinformatics* 29 3135–3142. 10.1093/bioinformatics/btt554 24064423 PMC5994942

