# Supplementary material for: DeepT3_4: A Hybrid Deep Neural Network Model for the Distinction Between Bacterial Type III and IV Secreted Effectors
Source: Front Microbiol. 2021 Jan 21;12:605782. doi: 10.3389/fmicb.2021.605782 (PMC7858263; doi:10.3389/fmicb.2021.605782)
Supplement: Supplementary file 1 [file Data_Sheet_1.docx]

Supplementary Material

DeepT3_4: A Hybrid Deep Neural Network Model for the Distinction between Bacterial Type III and IV Secreted Effectors

Lezheng Yu ^1^, Fengjuan Liu^2^, Yizhou Li^3^, Jiesi Luo^4*^ and Runyu Jing^3*^

^1^School of Chemistry and Materials Science, Guizhou Education University, Guiyang 550018, China

^2^School of Geography and Resources, Guizhou Education University, Guiyang 550018, China

^3^College of Cybersecurity, Sichuan University, Chengdu 610065, China

^4^Department of Pharmacology, School of Pharmacy, Southwest Medical University, Luzhou 646000, China

*** Correspondence:**Jiesi Luo

[ljs@swmu.edu.cn](mailto:ljs@swmu.edu.cn)

Runyu Jing

[jingryedu@gmail.com](mailto:jingryedu@gmail.com)


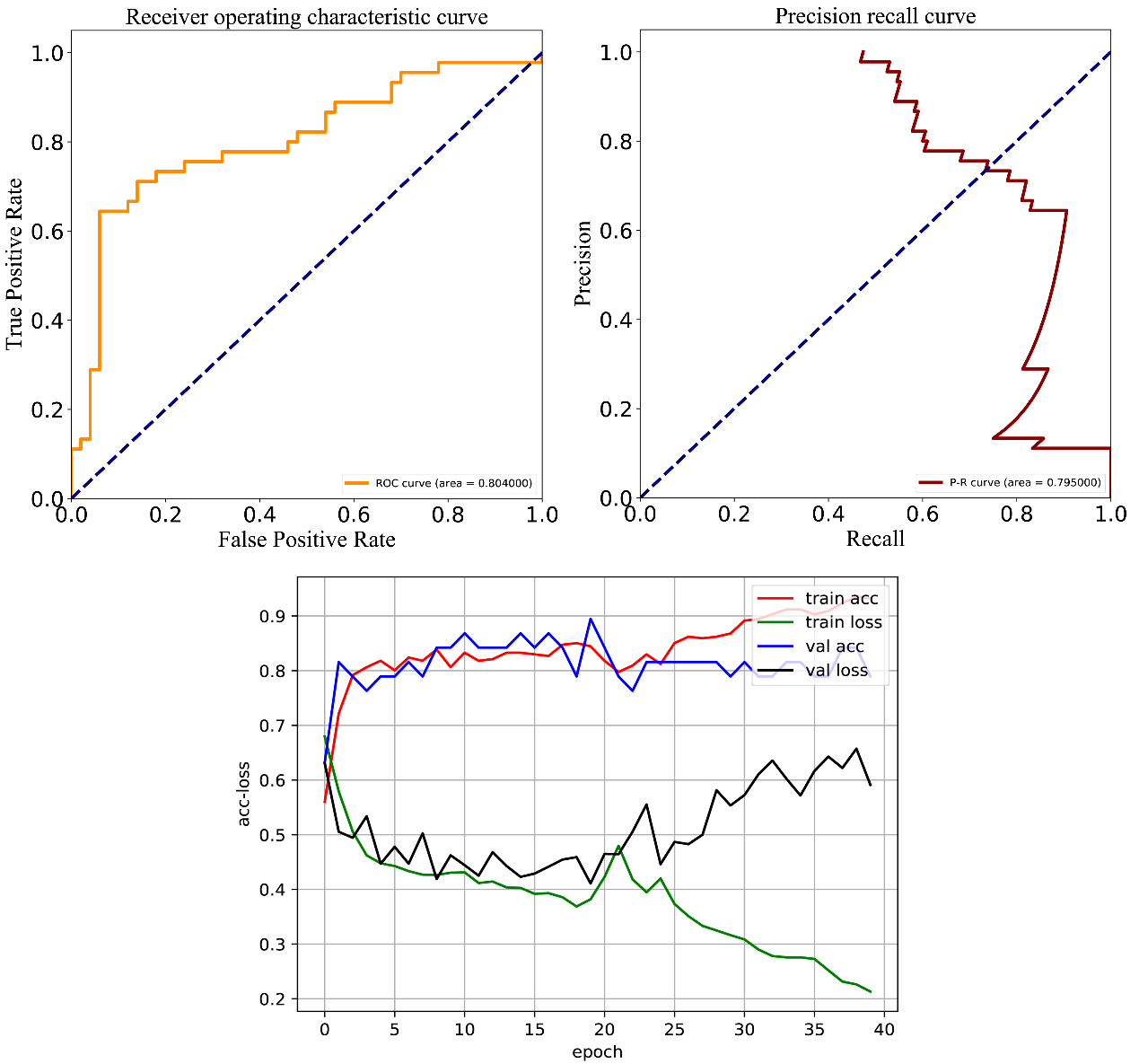


**Figure S1.** ROC, P-R and acc-loss curves generated by autoBioSeqpy tool for the RNN model on the test dataset.


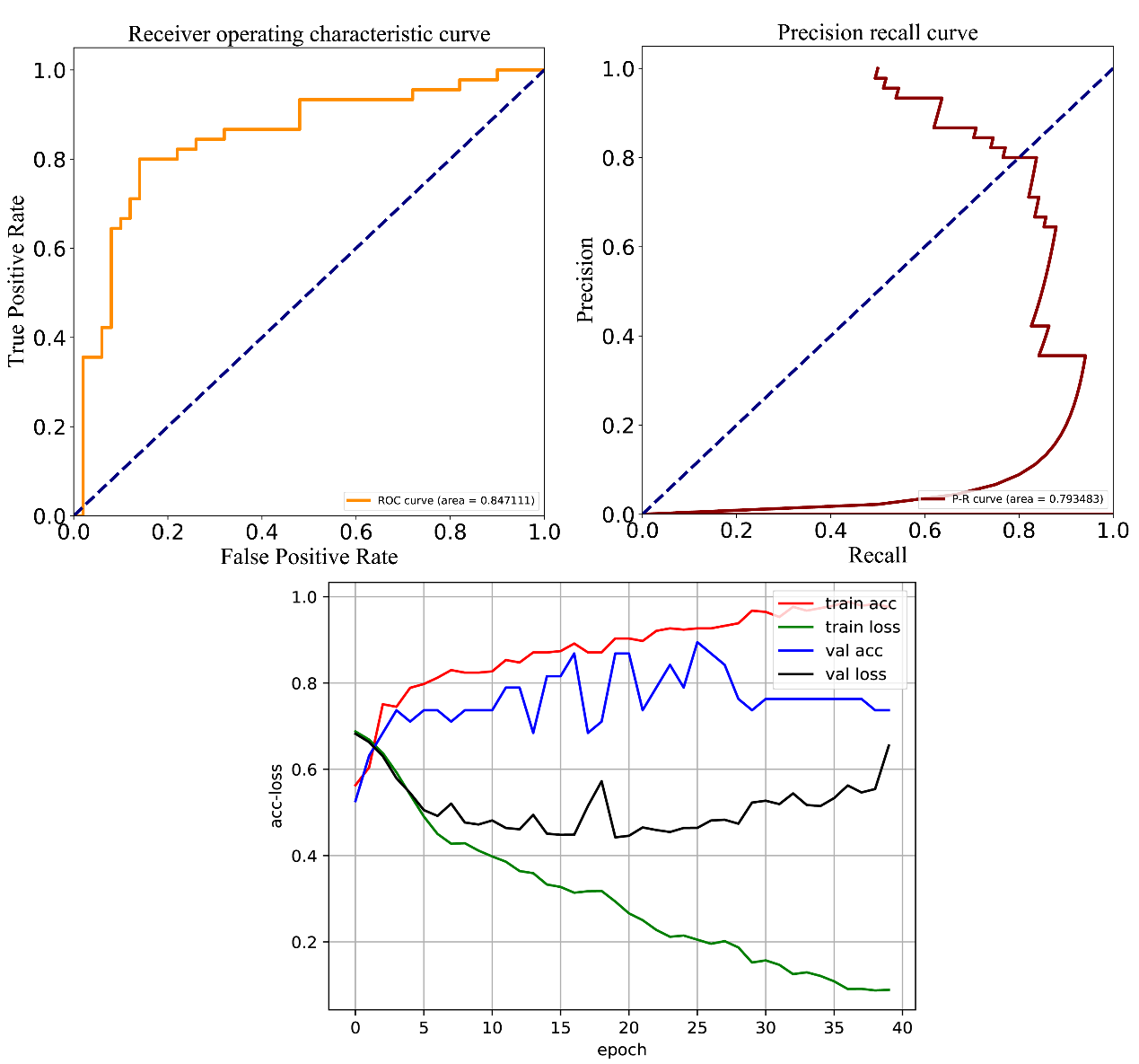


**Figure S2.** ROC, P-R and acc-loss curves generated by autoBioSeqpy tool for the DNN (AAC+DC) model on the test dataset.


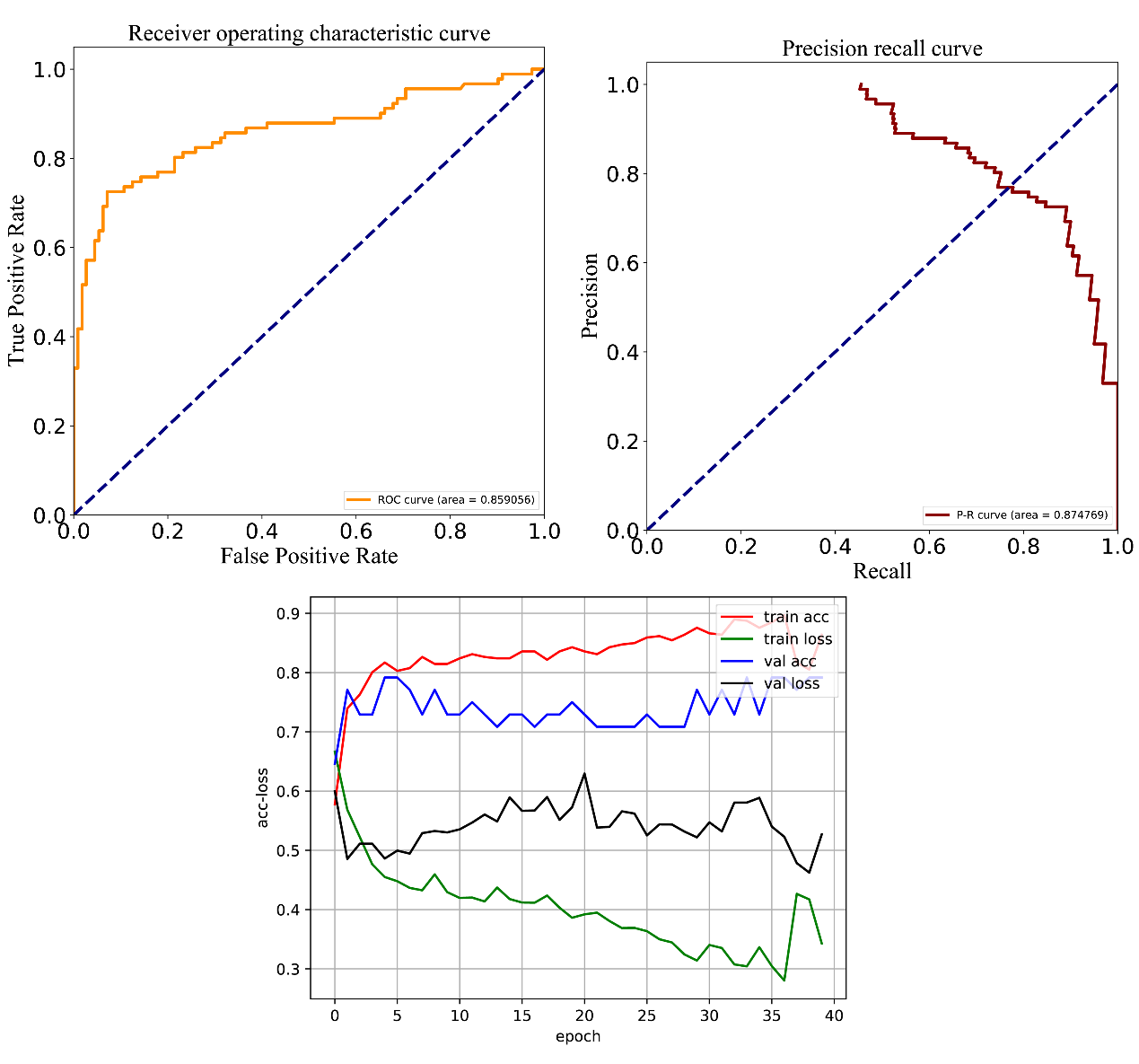


**Figure S3.** ROC, P-R and acc-loss curves generated by autoBioSeqpy tool for the RNN model on the independent test set.


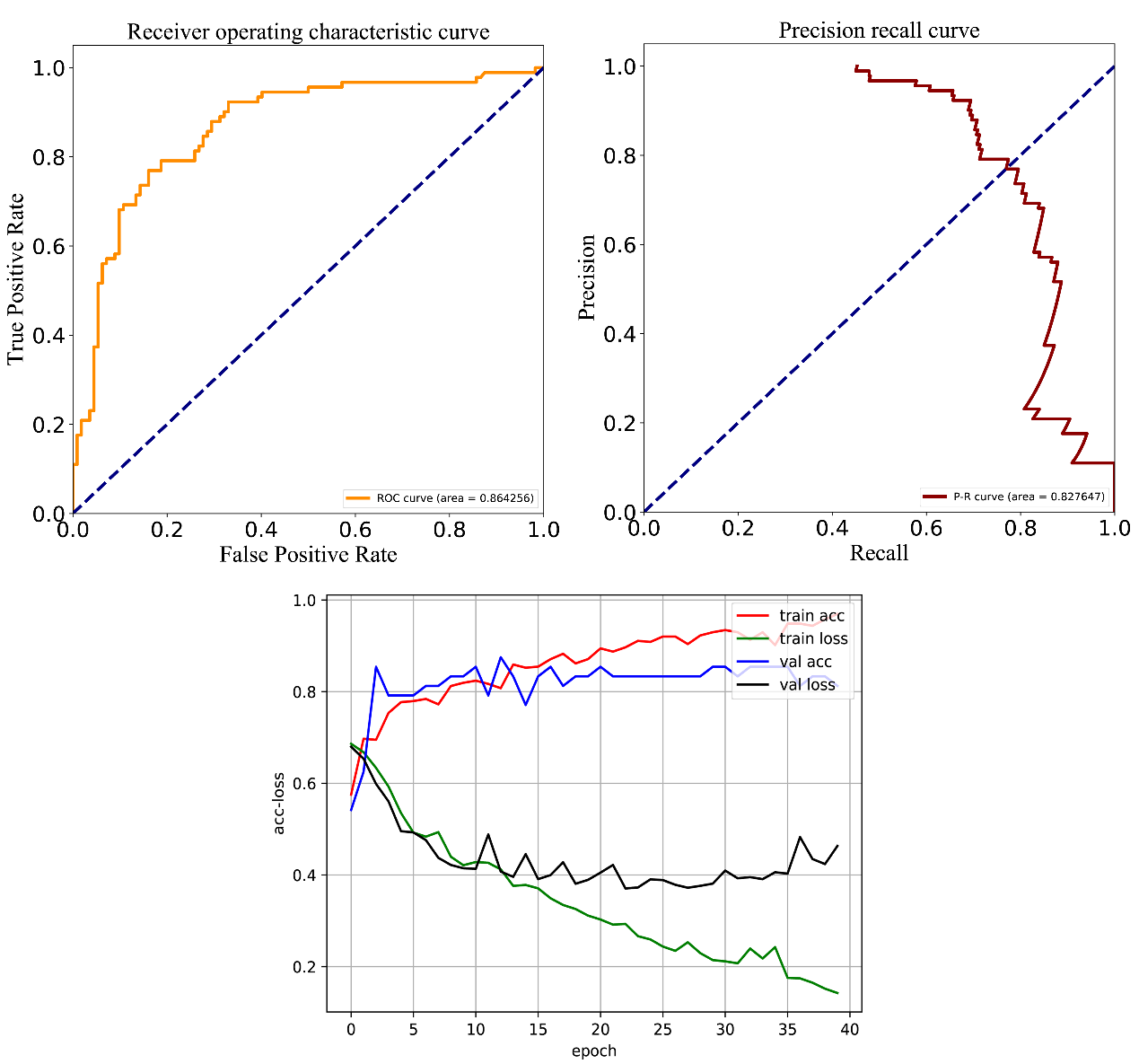


**Figure S4.** ROC, P-R and acc-loss curves generated by autoBioSeqpy tool for the DNN (AAC+DC) model on the independent test set.


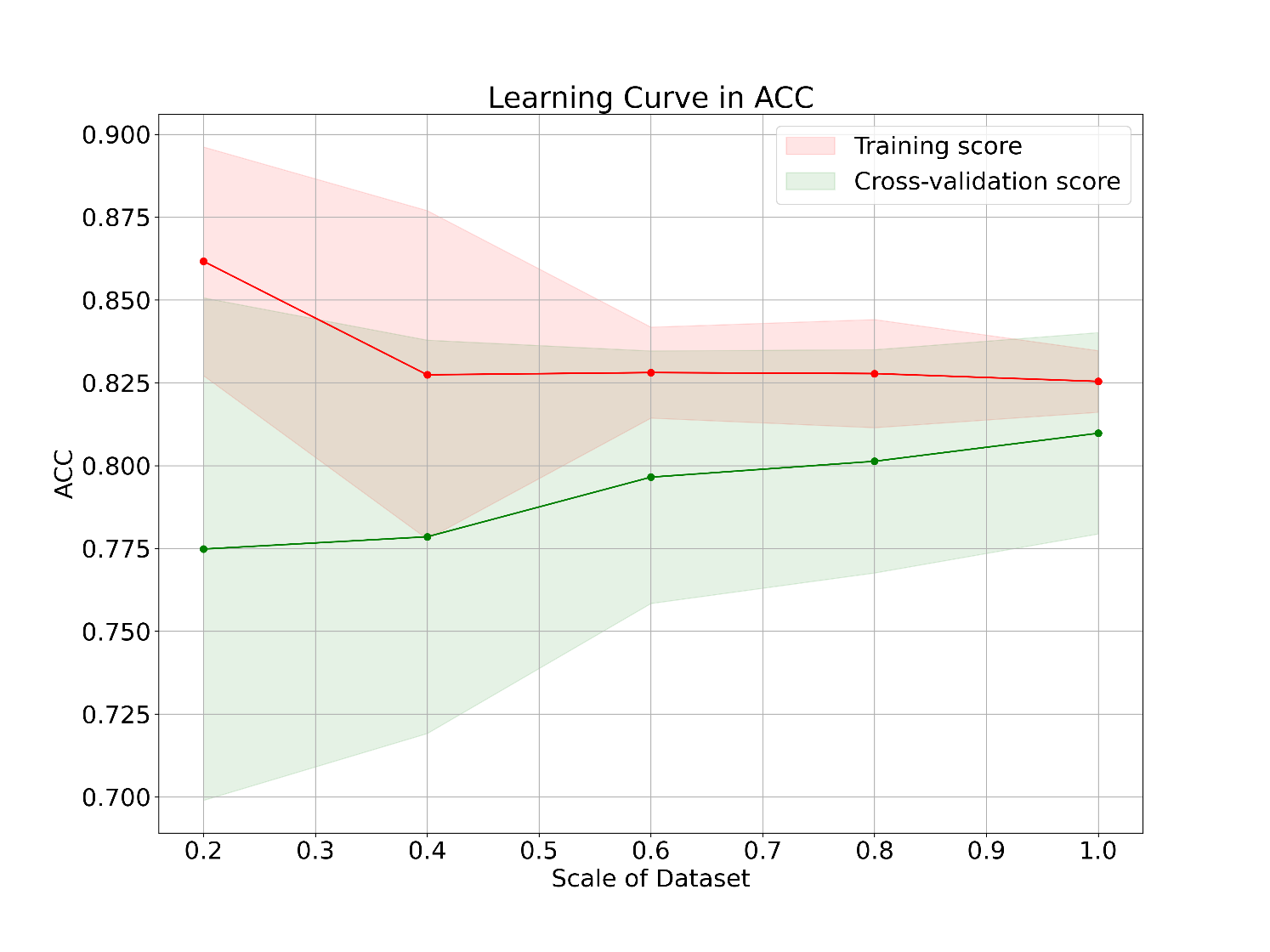


**Figure S5.** The learning curve for DeepT3_4 model using the ACC score.


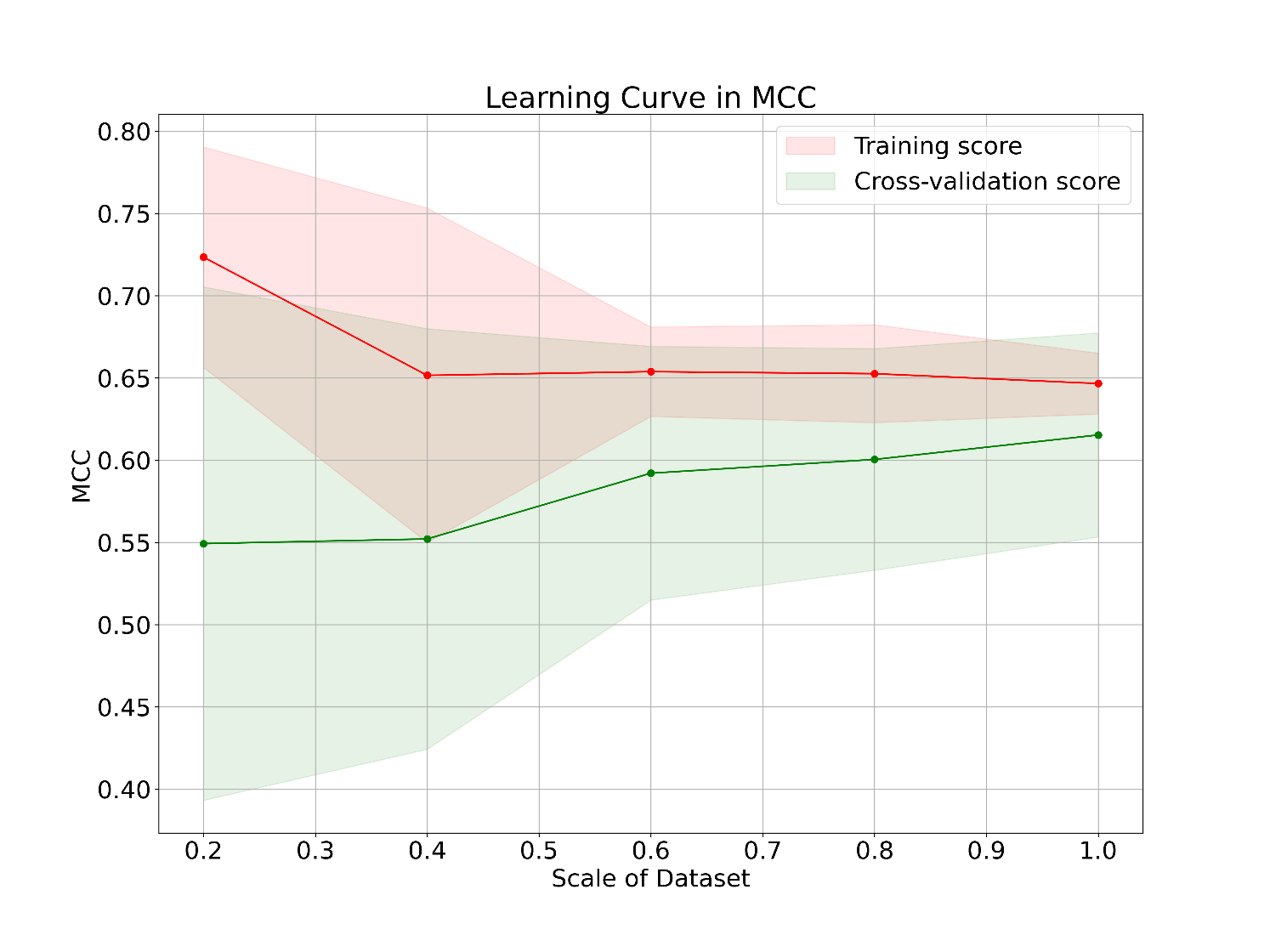


**Figure S6.** The learning curve for DeepT3_4 model using the MCC score.

| **Table S1.** The Benchmark and independent test datasets of T3SEs and T4SEs | | | | | | | |
| --- | --- | --- | --- | --- | --- | --- | --- |
| Benchmark dataset of T3SEs (211) | | | | | | | |
| Q156B7 | O84378 | Q9Z8G9 | C0SPQ2 | K2SG17 | D4SSF7 | O84144 | Q89TX4 |
| B5R8S6 | O84718 | Q9Z780 | Q2QHF9 | Q52473 | H6UWS2 | WP_030419593.1 | Q8ZNP4 |
| Q663L9 | D2TRA0 | D0ZXR5 | O84290 | P33548 | Q9Z8L3 | O84226 | F3IJ53 |
| EKJ11176.1 | A2S1Q7 | Q52430 | Q4ZX82 | Q63K40 | A1KWP8 | O84723 | Q4G4D0 |
| B7UMC8 | WP_009873285.1 | Q9Z8Z8 | Q84CS5 | Q01099 | Q7NUX3 | Q9FCY7 | O84625 |
| Q9I788 | D4HWL8 | CAA12191.1 | B0B9M4 | C5IZN1 | A9MG90 | A4PDT6 | F2ZE69 |
| Q46210 | Q57KZ6 | G9L9K6 | C0SPQ0 | O84700 | Q4ZX47 | D4SZP4 | Q9Z7E2 |
| Q4ZYH0 | Q58I88 | A2S1Q3 | P74851 | O84648 | F3E4M8 | D2TZ32 | Q3BRD8 |
| O84085 | D0ZPH9 | Q8XYE3 | Q9Z8P7 | Q3YTN8 | A1JQ83 | Q989P8 | O87327 |
| O69196 | C0SPQ1 | D0ZI38 | S6JQB0 | F2ZUJ4 | S3MY93 | Q3BSU7 | O34208 |
| Q8XTK9 | Q9AN16 | P0A1K5 | Q04640 | Q56019 | D2TK72 | Q87W42 | B7UNX4 |
| Q89N83 | Q881L7 | S6WSJ7 | O85345 | Q57P56 | CAA80362.1 | Q824H6 | P40699 |
| H9L477 | CAP03784.1 | Q8ZQC8 | Q9RBS2 | O84855 | P40296 | D2TTX7 | Q8RP17 |
| Q886L1 | Q9Z7W9 | Q9L6W3 | D8P4R3 | F3ICX9 | Q2T711 | K2TQ10 | K0GGG6 |
| D4HWC7 | Q7P1B7 | D2TI15 | Q63K50 | Q87P35 | B9A810 | WP_010875097.1 | Q9Z8X8 |
| O84856 | P0CZ04 | P68590 | Q888Y1 | C0SPP7 | D2AJU3 | D4SUA6 | Q6R8C3 |
| D0ZDK9 | D2TML3 | Q9REZ7 | Q989M4 | O84056 | P55724 | A0FKE5 | A2S1Q1 |
| Q3BY60 | Q79UN8 | Q9RBS0 | Q4ZXP9 | Q889A9 | Q6LAD6 | Q9R803 | D2TKG4 |
| Q5EN61 | P33546 | A9ZNF2 | B7UMR0 | P0CL47 | O33680 | O84462 | O84163 |
| B2TSV7 | WP_010883763.1 | B7UR62 | Q87X57 | P27474 | Q87W46 | Q52481 | E7PP21 |
| O30916 | C8TNS7 | P18013 | P28764 | Q9Z7K3 | Q9JP32 | Q8ZMY7 | P11437 |
| D2TZV2 | O84743 | O30783 | Q7WRZ5 | Q882F0 | Q9Z861 | Q3BYL8 | Q8ZMI3 |
| O84623 | K2RW35 | Q63K38 | D2TT38 | D2TKG5 | F3IP82 | D2AJI2 | Q87GF9 |
| Q9Z8P6 | D2TZ34 | C4PL71 | Q8ZN18 | H9L486 | O85666 | Q8XQE6 | A9MJD1 |
| Q9Z7J8 | D2AJ54 | Q3BW96 | Q9Z7Q6 | O84883 | B4SX34 | P0AE20 | A2I8A1 |
| O84436 | CBH40896.1 | Q08678 | Q88AB8 | B0B9M3 | Q326N4 | Q8Y125 | Q7VWI4 |
| Q989R4 | D2AJ70 | P37131 |  |  |  |  |  |
| Benchmark dataset of T4SEs (263) | | | | | | | |
| NP_819902.2 | NP_820552.1 | NP_820526.1 | Q5ZYT6 | WP_010948417.1 | SS01923 | Q5ZUX5 | Q5ZXN6 |
| Q5ZU59 | Q5ZYH0 | Q5ZSD5 | AFK10355.1 | Q5ZSK8 | WP_010947682.1 | WP_011996917.1 | Q5ZR83 |
| NP_821027.2 | Q5ZXD5 | P37033 | Q5ZYD6 | Q5ZYG7 | Q5ZUV5 | Q5ZVF1 | WP_010945807.1 |
| Q5ZRJ3 | Q5ZVE4 | Q5ZSU4 | SS01525 | Q5ZXT9 | Q5ZZ40 | Q5ZUV7 | NP_819448.1 |
| WP_012220016.1 | Q5ZUX9 | Q5ZYK7 | Q5ZZ16 | Q5ZYR7 | Q5ZWZ3 | NP_066749.1 | Q5ZU83 |
| NP_819814.1 | Q5ZY89 | Q5ZTK1 | WP_010948394.1 | Q5ZSC8 | Q5ZUV0 | NP_821048.1 | Q5ZRX6 |
| NP_819221.1 | NP_820731.1 | Q5ZYS7 | Q5ZUY1 | SS01911 | Q5ZTB4 | Q5ZYG9 | WP_012220000.1 |
| Q5ZWF6 | Q5ZUV8 | Q5ZZ76 | WP_010947674.1 | NP_820802.1 | WP_010947933.1 | WP_005771199.1 | NP_819950.2 |
| NP_819656.1 | NP_820618.1 | Q5ZVT4 | Q5ZR84 | WP_012220080.1 | Q5ZWA2 | Q5ZTD9 | Q5ZSU1 |
| Q5ZY53 | WP_011114402.1 | NP_820539.1 | WP_010931651.1 | WP_002966824.1 | Q5ZXJ8 | Q5ZZI0 | WP_012219989.1 |
| SS01722 | NP_820212.1 | Q5ZUB9 | Q5ZT43 | WP_005771948.1 | NP_819484.1 | Q5ZVT5 | NP_819338.1 |
| WP_011181145.1 | NP_820749.1 | Q5ZRQ4 | Q5ZZ03 | WP_011109645.1 | Q5ZTL2 | WP_012220693.1 | Q5ZTL7 |
| Q5ZTG3 | Q5ZRK2 | Q5ZSH6 | WP_010945931.1 | NP_819665.1 | WP_010974921.1 | Q5ZSG2 | Q5ZUX2 |
| Q29ST3 | Q5ZRT5 | NP_820668.1 | NP_820668.1 | YP_002332980.1 | Q5ZUV9 | Q5ZUB7 | Q5ZU46 |
| Q5ZV52 | SS01693 | WP_005769508.1 | Q5ZUE7 | Q5ZWE0 | WP_005772222.1 | Q5ZWH7 | NP_819427.1 |
| NP_819096.1 | Q5ZS27 | Q5ZZH2 | SS01681 | Q5ZSM7 | Q5ZSR1 | Q5ZUG2 | WP_011181142.1 |
| Q5ZWI4 | Q5ZT14 | Q5ZX07 | Q5ZU30 | NP_819070.1 | SS01956 | NP_820443.1 | Q5ZVF7 |
| Q5ZUK0 | Q5ZUK1 | Q5ZUS5 | WP_010974922.1 | WP_002966455.1 | WP_002964382.1 | NP_820305.1 | Q5ZSU6 |
| Q5ZZK2 | Q5ZTK6 | Q5ZSZ1 | NP_819802.1 | Q5ZWH4 | Q5ZWB9 | NP_819463.1 | Q5ZV62 |
| Q5ZWW6 | WP_010947885.1 | WP_010957471.1 | Q5ZRQ0 | Q5ZYH1 | Q5ZVF2 | WP_010945988.1 | Q5ZU75 |
| Q5ZU61 | WP_010945803.1 | Q5ZZA4 | YP_001961089.1 | NP_059824.1 | Q5ZSL2 | Q5ZTC3 | Q5ZZC1 |
| Q5ZWB4 | Q5ZWH9 | Q5ZT21 | Q5ZU48 | Q5ZX35 | Q5ZRQ1 | Q5ZRX7 | WP_011181138.1 |
| Q5ZSW0 | Q5ZV89 | NP_819821.1 | Q5ZU36 | Q5ZXW0 | WP_010946018.1 | Q5ZVX6 | Q5ZRS4 |
| WP_010948419.1 | Q5ZZ01 | Q5ZZ20 | Q5ZUL7 | Q5ZV42 | Q5ZZ71 | Q5ZWE1 | Q5ZYS6 |
| Q5ZYJ6 | Q5ZSL9 | WP_011181144.1 | Q5ZW60 | WP_012220629.1 | WP_011997383.1 | Q5ZZD1 | Q5ZUX6 |
| WP_010948500.1 | Q5ZRK7 | Q5ZUH6 | YP_002333001.1 | Q5ZSJ2 | WP_011114288.1 | SS01719 | Q5ZV21 |
| Q5ZZF1 | Q5ZWE8 | Q5ZT79 | Q5ZYT5 | Q5ZSE2 | Q5ZRQ2 | Q5ZUJ5 | Q5ZWG1 |
| Q5ZV03 | Q5ZY54 | Q5ZU45 | WP_010946453.1 | Q5ZSK6 | Q5ZZI9 | Q5ZRH5 | Q5ZZ30 |
| Q5ZZ85 | Q5ZX12 | WP_011996490.1 | Q5ZSR5 | Q5ZVX2 | Q5ZSI8 | Q5ZT91 | WP_010931648.1 |
| Q5ZYD5 | WP_011450840.1 | Q5ZU44 | Q5ZVZ8 | Q5ZVI7 | Q5ZYI2 | Q5ZWD1 | WP_002963813.1 |
| Q5ZUY2 | WP_011114156.1 | WP_010948288.1 | Q5ZSL3 | Q5ZRS6 | Q5ZSU2 | Q5ZZB5 | Q5ZUP1 |
| Q5ZZ51 | Q5ZTJ7 | Q5ZSV5 | Q5ZST2 | Q5ZSB6 | Q5ZXU7 | Q5ZYD3 | NP_862934.1 |
| WP_012220099.1 | Q5ZTJ5 | Q5ZU60 | WP_011216046.1 | Q5ZZG4 | Q5ZWW7 | WP_012220155.1 |  |
| Independent test set of T3SEs (91) | | | | | | | |
| H9L446 | D8P480 | Q05608 | Q326T5 | Q84CS9 | Q9F0H3 | E5G0U5 | SS01214 |
| Q89TT5 | A1JUA6 | A1JUA9 | K2T9V0 | B7UMA0 | Q87P38 | P13835 | D0ZIB5 |
| Q887B7 | A1JU78 | A1JU65 | O84947 | Q48BE0 | D1MWR4 | Q2QCI9 | Q52497 |
| Q08242 | O84449 | D2TKE1 | C8BNW8 | AJK93307.1 | K2T7W2 | O84232 | O84107 |
| Q9L6W4 | B7UM94 | Q9Z877 | Q6WEG4 | K2TF22 | D2TKD7 | D2TJZ3 | E1WAC6 |
| A2S1Q9 | Q48B68 | Q87P32 | Q87V79 | Q9FD10 | Q89TP9 | Q2AC60 | D2TKF1 |
| Q9AMW4 | D2TRX8 | Q9RBS1 | ACT71547.1 | O84554 | P55704 | WP_005479246.1 | A9R9H4 |
| Q7N439 | Q52420 | O84616 | Q60236 | K2SVJ4 | Q7PC62 | C8BNV1 | Q2NVH6 |
| C0SPQ6 | F3DQ26 | Q7PC42 | NP_052408.1 | C4PL72 | Q4G4C8 | Q83XF9 | O84342 |
| O84854 | O84871 | D2TKH5 | B0B9M5 | Q89TW7 | F2ZR74 | Q9Z6N8 | P40613 |
| Q88BQ2 | Q9Z785 | Q9Z8N0 | Q87W65 | O84869 | O84120 | O84944 | O84235 |
| P37132 | D2TZ31 | Q6VE93 |  |  |  |  |  |
| Independent test set of T4SEs (112) | | | | | | | |
| Q5ZWG4 | Q5ZU39 | Q5ZWD3 | NP_820705.1 | Q5ZWY9 | WP_011216437.1 | NP_819899.1 | Q5ZU49 |
| Q5ZRP8 | WP_012569937.1 | Q5ZTL4 | Q5ZWD7 | Q5ZRA8 | Q5ZU58 | Q5ZRE6 | Q5ZUS4 |
| WP_010947428.1 | Q5ZSJ4 | Q5ZWH6 | Q5ZWW5 | WP_010947059.1 | Q5ZRR5 | Q5ZSY4 | WP_010946171.1 |
| WP_010946263.1 | WP_010948114.1 | Q5ZTE7 | Q5ZZ39 | Q5ZYV1 | SS01678 | Q5ZWK2 | Q5ZS91 |
| Q5ZWY8 | Q5ZT54 | NP_820409.1 | Q5ZS82 | Q5ZRJ7 | Q5ZSH8 | Q5ZSV9 | Q5ZUA0 |
| Q5ZVJ1 | WP_011996398.1 | Q5ZW15 | Q5ZU22 | Q5ZVF6 | Q5ZSQ2 | Q5ZUS6 | Q5ZYH9 |
| WP_010974923.1 | Q5ZXN5 | Q5ZU43 | Q5ZWH5 | NP_957603.1 | Q5ZRP9 | Q5ZZD0 | Q5ZYV7 |
| Q5ZSQ6 | YP_001961003.1 | WP_002965067.1 | Q5ZUY8 | Q5ZXT6 | Q5ZYJ7 | Q5ZT06 | Q5ZZJ8 |
| Q5ZSS2 | Q5ZRH0 | Q5ZTG2 | Q5ZT65 | Q5ZSJ0 | Q5ZU55 | Q5ZVL4 | Q5ZVE9 |
| WP_002969602.1 | Q5ZSH9 | Q5ZTV8 | Q5ZVT6 | Q5ZTE6 | Q5ZTB8 | WP_010929491.1 | WP_000180783.1 |
| YP_001967606.1 | Q5ZYK8 | SS01691 | WP_011452831.1 | Q5ZT67 | Q5ZYW5 | Q5ZRJ6 | Q5ZVN2 |
| Q5ZSS3 | Q5ZSV7 | WP_011996878.1 | Q5ZSN5 | Q5ZZ81 | SS01540 | Q5ZSG5 | Q5ZZA8 |
| Q5ZSJ1 | Q5ZWX1 | Q5ZU59 | Q5ZUP2 | SS01627 | SS01724 | WP_010948033.1 | Q5ZWC7 |
| ALB19409.1 | Q5ZSZ4 | Q5ZV00 | Q5ZSH1 | Q5ZRN6 | Q5ZYX1 | Q5ZSZ6 | Q5ZV17 |

| **Table S2**. The 5-Fold Cross-Validation Details in the Benchmark dataset | | | | | | |
| --- | --- | --- | --- | --- | --- | --- |
| Fold set | Method | ACC (%) | *F*-value (%) | Recall (%) | PRE (%) | *MCC* |
| 1 |  | 87.2 | 85.0 | 81.4 | 89.2 | 0.743 |
| 2 |  | 85.0 | 82.2 | 77.7 | 87.6 | 0.698 |
| 3 | DeepT3_4 | 81.0 | 79.5 | 83.3 | 76.1 | 0.622 |
| 4 |  | 85.0 | 82.3 | 79.0 | 86.1 | 0.697 |
| 5 |  | 81.4 | 78.4 | 75.7 | 81.7 | 0.626 |
| Average |  | 83.9 ± 2.6 | 81.5 ± 2.6 | 79.4 ± 3.0 | 84.1 ± 5.3 | 0.677 ± 0.052 |

**Table S3.** Performance Comparisons of the RNN&DNN model trained by using the N-terminal, C-terminal, and full protein sequences.

| Sequence | ACC (%) | *F*-value (%) | Recall(%) | PRE (%) | MCC |
| --- | --- | --- | --- | --- | --- |
| N-terminal | 77.5 | 74.7 | 74.1 | 75.3 | 0.544 |
| C-terminal | 71.0 | 68.9 | 71.6 | 66.4 | 0.419 |
| Full | 82.3 | 80.5 | 81.2 | 80.0 | 0.645 |
